# Development and Psychometric Validation of the Mental Health-Related Barriers and Benefits to EXercise (MEX) Scale in Healthy Adults

**DOI:** 10.1186/s40798-023-00555-x

**Published:** 2023-02-23

**Authors:** Madeleine L. Connolly, Stephen C. Bowden, Michaela C. Pascoe, Nicholas T. Van Dam

**Affiliations:** 1 Melbourne School of Psychological Sciences, The University of Melbourne, Level 12, Redmond Barry Building, Parkville, Melbourne, 3010 Australia; 2 Centre for Clinical Neurosciences and Neurological Research, St Vincent’s Hospital Melbourne, Melbourne, 3065 Australia; 3 Institute for Health and Sport, Victoria University, Melbourne, 3011 Australia

**Keywords:** Anxiety, Depression, Stress, Barriers, Benefits, Exercise

## Abstract

**Background:**

Physical exercise has been shown to reduce anxiety and depression symptoms, the most common mental health disorders globally. Despite the benefits of exercise in anxiety and depression, the symptoms of these disorders may directly contribute to a lack of engagement with exercise. However, mental health-related barriers and benefits to exercise engagement have not been addressed in quantitative research. We introduce the development and psychometric validation of the Mental health-related barriers and benefits to EXercise (MEX) scale.

**Methods:**

Three samples were collected online prospectively (sample 1 *n* = 492; sample 2 *n* = 302; sample 3 *n* = 303) for scale refinement and validation with exploratory and confirmatory factor analysis. All participants were generally healthy adults, aged 18–45, and had no history of severe mental illness requiring hospitalization and no physical disability impacting over 50% of daily function.

**Results:**

We identified a 30-item, two-factor model comprising 15 barrier and 15 benefit items. Overall model fit was excellent for an item-level scale across the three samples (Comparative Fit Index = 0.935–0.951; Root-Mean-Square Error of Approximation = 0.037–0.039). Internal consistency was also excellent across the three samples (*α* = 0.900–0.951). The barriers subscale was positively correlated with symptoms of anxiety, depression and stress, and negatively correlated with measures of physical activity and exercise engagement. The benefits subscale was negatively correlated with symptoms of anxiety, depression and stress, and positively correlated with measures of physical activity and exercise engagement.

**Conclusion:**

The MEX is a novel, psychometrically robust scale, which is appropriate for research and for clinical use to ascertain individual and/or group level mental health-related barriers and benefits to exercise.

**Supplementary Information:**

The online version contains supplementary material available at 10.1186/s40798-023-00555-x.

## Background

Anxious and depressive disorders are the most common mental disorders among adults worldwide and leading causes of disability [1]. As a result, the promotion of mental health and the prevention of mental health disorders is paramount. Exercise, defined here as effortful physical activity in leisure time, is a promising approach for the prevention and treatment for symptoms of both anxiety and depression at both clinical and subclinical levels [2]. As a treatment option, exercise requires motivation and action for uptake and adherence. However, factors such as a pervasive lack of motivation and fear-based avoidance—key clinical features of depression and anxiety, respectively—may potentially create barriers to engagement in leisure-time exercise for individuals who experience such symptoms [3, 4]. Other symptoms, such as concerns about appearance, sensitivity to somatic signals (e.g. heart rate, respiration), and avoidance, may also critically interact with uptake of and adherence to exercise. Furthermore, there are broader psychological benefits to exercise beyond the overall score reduction on anxiety or depressive scales that are typically reported for clinical [5] and subclinical populations [2]. Thus, an important bidirectional relationship may apply. While exercise may reduce or improve specific symptoms of anxiety and depression, the experience of the symptoms themselves may prevent people from exercising.

Measurement of mental health-related barriers to, and benefits of exercise engagement is crucial to understanding the bidirectional relationship and, for achieving appropriate, tailored exercise prescriptions in individuals with symptoms of anxiety and depression. It is important to acknowledge a distinction between exercise and physical activity (PA). Whilst exercise refers to effortful physical activity in leisure time specifically, PA refers to all activity across life domains including leisure time, but also including any movement associated with occupation, transport, housework, and any other non-leisure-time domains. Although many types of PA are beneficial for physical health, leisure-time exercise is particularly beneficial for mental health [6]. It is also important to acknowledge both the barriers and benefits to exercise engagement during early adulthood, as this period is marked by a decline in PA and exercise engagement [7]. Furthermore, early adulthood is also a common time for the onset of mental disorders, with recent estimates of median ages of onset of anxiety and mood disorders being 17 and 31, respectively [8].

Current quantitative models of benefits and barriers to exercise are based on scales that are intended for the general adult population and cover a wide range of general, logistical and physical barriers and benefits to exercise, without a distinct focus on individual mental health [9]. Consequently, these models are missing specific features of anxiety- and depression-like symptoms that may be relevant to exercise behaviours, in both clinical and subclinical populations. In essence, quantitative research into the barriers and benefits to exercise engagement only assesses a small subset of psychologically relevant items, always combined with a much larger list of physical or logistical barriers and benefits to exercise [3, 9–11]. Meanwhile, qualitative research has demonstrated that the actual experience of anxious and depressive symptoms appear to become barriers to exercise engagement, whilst the relief of these symptoms is a benefit of exercise engagement [12, 13]. Despite such qualitative evidence, specific quantitative assessment of mental health-related barriers and benefits to exercise is critical to understanding how symptoms are related to exercise.

### Mental Health-Related Benefits of Exercise

In their meta-analysis of the benefits and barriers to exercise for depressive and mood disorders, Glowacki et al. report improved mood, better attention and alertness, and better stress management as major benefits from exercise [3]. Similarly, Searle and colleagues’ qualitative evidence from a sample of 33 depressed individuals suggests that positive distraction from depressive symptoms and providing a sense of purpose are key benefits of exercise [4]. Improvements in energy, a positive feeling of ‘pushing oneself’, a sense of regaining pleasure, and increases in self-confidence, self-efficacy, body image, and autonomy are also factors reported by participants with depression and following exercise engagement in qualitative research [4, 13]. Accordingly, several key symptoms of depression (including low mood, high stress, low energy, anhedonia, low self-confidence and low self-efficacy) are improved with exercise.

Qualitative evidence also points to psychologically relevant benefits of exercise for those with anxiety disorders, relating to both the alleviation of anxious symptoms and of general negative affect. Mason et al. [12] interviewed a heterogeneous sample of individuals with symptoms of at least one anxiety or related disorder (e.g. obsessive–compulsive disorder, specific phobia). Following exercise engagement, they observed decreases in frequency and severity of worry (especially among those with generalized anxiety), social benefits (particularly by those with social anxiety symptoms), and the perception that exercise was a useful distraction from anxious thoughts. Finally, exercise was also reported to reduce overall stress levels among individuals with anxiety and depression [3, 4]. It is important to note that there are many psychological and mental health-related benefits of exercise, and the benefits reported here are not exhaustive. Further information on mental health-related benefits of exercise is available in Mikkelsen et al.’s review [14].

### Mental Health-Related Barriers to Exercise

Barriers to exercise which mirror depressive and anxious symptoms appear frequently in qualitative data from clinical samples, and to a smaller extent in quantitative data representing subclinical samples. Such barriers are not only limited to the more commonly cited barriers of lack of motivation and fear-based avoidance, but also include a variety of transdiagnostic mental health-related barriers to exercise [3, 4].

In quantitative research for non-mental health specific barriers to exercise, some of the most highly endorsed barriers in the general population samples were: low confidence when sad or distressed (75% endorsement in a sample of 138), fatigue (69%, total sample *n* = 203), low mood (65%, total sample *n* = 102), feeling unwell (60%, total sample *n* = 101), and feeling too shy or embarrassed (36%, total sample *n* = 101) [3]. Interestingly, of these five barriers, three (low confidence, fatigue, and low mood) directly mirror symptoms of depression, and one (feeling too shy or embarrassed) mirrors social anxiety. Similarly, in qualitative research among individuals diagnosed with depressive disorders, there are many reports of barriers to exercise which mirror depressive symptoms, such as low motivation and low confidence [3, 4]. The qualitative research also points to further barriers to exercise in clinical depression samples that are more like anxious or transdiagnostic symptoms, such as guilt, stress, fear of social interaction, and poor body image [4].

Exercise-related anxiety (i.e. anxiety specific to the activity of exercising) has also been reported as a barrier to exercise. For individuals with high levels of symptomatic anxiety, qualitative interviews indicate that specific fears or anxieties can negatively affect exercise participation and become barriers to exercise. For example, participants with social anxiety report fearing social evaluation during exercise, and consequently avoid exercise behaviours [12]. For participants with general anxiety, fears such as injury or pain were also key barriers. Additionally, psychosomatic sensations of anxiety during exercise were also reported as barriers to future exercise sessions by some with anxiety [12]. Transdiagnostic symptoms of low self-confidence, fatigue, life stress, and worries of lack of exercise knowledge or technique have also been noted as barriers to exercise for individuals with high levels of anxious symptoms [12].

As with the benefits of exercise, the mental health-related barriers to exercise reported by individuals with depressive and anxious symptoms show considerable transdiagnostic overlap. Such barriers can be labelled as low confidence, low mood, fatigue, stress, body image, worry, social anxiety, fear of injury, and lack of exercise knowledge and skills.

### The Novel Mental Health-Related Barriers and Benefits to Exercise Scale

The aim of this study was to develop and validate a novel scale with items specific to the barriers and benefits of physical exercise, relevant to mental health and psychological well-being. The scale was intended to assess the mental health-related barriers and benefits of exercise for individuals with both varied levels of anxious and depressive symptoms. The scale therefore aimed to quantify the psychological barriers and benefits to exercise in generally healthy adults without severe mental illness.

It was hypothesized that the *Mental health-related barriers and benefits to physical EXercise* (MEX) scale would comprise two main factors, a barriers subscale and a benefits subscale. Within each subscale, multiple lower-order factors were explored.

It was also hypothesized that the two subscales would have associations with measures of mental health, psychological well-being, and physical activity and exercise. The barriers subscale was predicted to have positive correlations with mental ill health, and negative correlations with psychological well-being, and with physical activity and exercise. Meanwhile, the benefits subscale was predicted to have a negative correlation with mental ill health, but positive correlations with psychological well-being, and with physical activity and exercise.

## Methods

### Participants and Design

The present study employed data from two data sets, the second of which was randomly divided, to create three samples in total. Recruitment of all participants was aimed at the general population, with the aim of recruiting generally healthy adults without severe mental illness. Sample 1, the initial scale development data set, was procured using Amazon’s Mechanical Turk (Mturk) in 2020 (final *n* = 492). The second data set consisted of participants recruited via Prolific in 2021 (final *n* = 605). This second data set was split (sample 2: *n* = 302; sample 3: *n* = 303) so that Sample 2 data could be used in scale refinement, and Sample 3 data remained a separate validation sample. Given the difficulty of ascertaining power for factor analysis [15], we aimed to recruit as many participants as possible in both stages of recruitment.

In the first data set, geographical location was restricted to US-based participants to mitigate potential confounds associated with geo-cultural differences in item interpretation. In the second data set, geographical location was restricted to residents of the USA, the UK, Australia, and New Zealand; representation was expanded to allow for greater ecological validity. All participants across each data set were between 18 and 45 years of age (to minimize age-related variation in activity level), and were deemed eligible on the basis of satisfying the following inclusion criteria: (1) no reported history of severe mental illness; (2) no self-reported physical disability perceived by participant to negatively impact > 50% of their daily function. Participants from each data set were also excluded during data cleaning processes if they did not satisfy the attention check and careful responding criteria as listed below (see *Data Cleaning Procedure*). Full demographic information and data processing outcomes are described under *Results.* Participants were asked about their experience of mental health symptoms, as well as their current activity levels. Upon completion of the total battery, participants were remunerated at a minimum rate of $10US per hour. The collection of data for both data sets was approved by the University of Melbourne Human Research Ethics Committee [HREC # 2056662], and all protocols conformed to the ethical standards outlined in the Declaration of Helsinki.

### Measures

#### The MEX Scale

The initial pool numbered 113 items (53 benefits, 58 barriers, 1 item for ‘benefit facilitation’, 1 item for ‘barrier interference’). A number of items for the Barrier and Benefits subscales in the MEX were inspired by psychologically relevant barrier and benefit items from prior quantitative research [9–11, 16–19]. Further, novel quantitative items were written by the authors with inspiration from qualitative research on depression [13], and on anxiety disorders [12]. Finally, a small number of novel items were also written by the authors with inspiration from informal piloting with 30 adults from the general population, and from the authors’ clinical knowledge and expertise. All 113 items were administered to sample 1. A subset of the MEX scale (48 items; 16 benefit items and 32 barrier items) was administered to sample 2/3.

#### Depression, Anxiety, and Stress Scales (DASS-42)

The DASS-42 is a 42-item scale designed to measure depression, anxiety, and stress on a continuum [20]. The DASS-42 has been validated previously by factor analysis and shows good reliability [20]. Each of the DASS-42 items is scored on a 4-point scale, in which higher scores indicate higher levels of depression, anxiety, or stress [20]. The DASS-42 was only administered to Sample 1. Each subscale showed a bimodal distribution in Sample 1.

#### The International Physical Activity Questionnaire—Short Form (IPAQ-SF)

The IPAQ-SF is a 7-item questionnaire designed to measure the weekly minutes of all physical activity and transform these activity minutes into metabolic equivalent units (METs) [21]. The IPAQ-SF has participants recall and report weekly hours and/or minutes of all physical activity, including work and home domains, as well as leisure-time activity. Participants reported the activity as either ‘vigorous’, ‘moderate’, or ‘walking’. Each category of intensity was then multiplied by the corresponding metabolic equivalent and then summed for a score of METs over one week, where higher METs indicate higher levels of activity. The IPAQ-SF was chosen here for its brevity in administration; however, it has previously shown weak external validity when correlated with objective accelometric measures, and self-report appears to be more prone to overestimation by those who are more sedentary [22]. The IPAQ-SF was only administered to Sample 1.

#### The Satisfaction With Life Scale (The SWLS)

The SWLS is a 5-item scale, designed to measure overall satisfaction with life [23]. The SWLS has been validated previously by factor analysis, and shows good external validity [23]. Each of the SWLS items is scored on a 6-point scale, in which higher scores indicate higher levels of life satisfaction and psychological well-being [23]. The measure was chosen as a counterbalance for the DASS-42, which only measures negative affect. The SWLS was only administered to Sample 1.

#### The Balanced Inventory of Desirable Responding Scale—Short Form (The BIDR)

The BIDR is a 16-item scale designed to measure the extent to which participants have bias in responses either in terms of a) impression management (8 items), or b) self-deceptive enhancement (8 items) [24]. The BIDR has been validated previously by factor analysis, and shows good reliability [24]. Each of the BIDR items is scored on a 5-point scale, in which higher scores indicate higher levels of either impression management or self-deceptive enhancement [24]. This scale was selected to assess the extent to which the MEX subscales would correlate with either measure of response bias. The BIDR was only administered to Sample 1.

#### Subjective Activity and Fitness Questions

All participants were presented with questions regarding how they would evaluate their own physical activity and fitness in terms of general sufficiency, in terms of their ideal levels of each, and each as compared to the average person of their age [25]. Such questions have previously shown high external validity and correlations with psychological measures such as distress and sleep [25]. Higher scores on subjective activity and fitness indicate higher perceived levels of activity and fitness. These questions were administered to both samples.

#### COVID-19 Impact Questions

All participants were asked questions to assess the potential negative impact and extent of any impact of the COVID-19 pandemic on their mental health, and exercise habits. These questions were administered to both samples.

#### The Mood and Anxiety Symptom Questionnaire-90 (MASQ-90)

The MASQ-90 is a 90-item questionnaire designed to measure: Anxious Arousal, Anhedonic Depression, and three types of Symptoms of General Distress: Depressive Symptoms, Anxious Symptoms, and Mixed Symptoms [26]. The MASQ-90 has been validated previously by factor analysis, and shows good reliability [26]. Each of the MASQ-90 items is scored on a 5-point scale, in which higher scores indicate higher levels of depression, anxiety, or distress [26]. The MASQ-90 was only administered to Sample 2/3. Each of the subscales was positively skewed in distribution for samples 2/3.

#### Leisure Time Sport and Exercise Survey (LTSES)

The LTSES is a brief scale designed to capture habitual physical activity across the domain of sport and exercise activity in leisure time [27]. Participants are asked to report their two most frequented sports or leisure-time activities (e.g. running, walking, or gym-based activity) and to report the intensity of each activity, and the time spent on each activity in terms of months per year and minutes per week. The overall score is transformed to a number between 0 and 4, in which 0 corresponds to no leisure-time exercise and 4 corresponds to a very high amount of leisure-time exercise [27]. The LTSES has been validated previously by factor analysis and shows good reliability [27]. The LTSES was only administered to Sample 2/3.

### Data Cleaning Procedure

Data were excluded casewise if it did not satisfy the following criteria: a recaptcha score of over 0.7 (Qualtrics recommended threshold), and passing all attention checks and basic writing and reading comprehension checks. The data were also screened for long string response outliers [28], high intra-individual response variability (IRV) [29], and multivariate outliers by Mahalanobis distance. Long string response outliers were judged by visual inspection of the long string response histogram per data set. High IRV was determined by the score corresponding to the 99^th^ percentile of IRV scores in a randomized simulation of each data set. Finally, the Mahalanobis distance outlier cut-off score was determined by the value corresponding to the 99.9th percentile of the chi-square distribution of Mahalanobis distances for the remaining participants in each sample after prior cases had been removed. Full details of data cleaning results for each sample are available in Additional file 1.

### Data Analysis Procedure

Data were analysed in R (version 4.1.0). For exploratory factor analysis (EFA), we used promax rotation and maximum likelihood estimation. For confirmatory factor analysis (CFA), we used weighted least squares mean and variance adjusted estimation, due to its robustness with ordered categorical items. Fit statistics were judged against the Hu and Bentler criteria for good fit: Tucker–Lewis Index (TLI) and Comparative Fit Index (CFI) above 0.95, Standardized Root-Mean-Squared Residual (SRMR) under 0.08, and Root-Mean-Square Error of Approximation (RMSEA) under 0.06 [30]. We note that these are general and conservative heuristics, which are better for parcel-level factor analysis; we therefore apply some leniency in the interpretation of our item-level analysis [31, 32].

Following factor analytic procedures, the reliability of the scale was determined using both alpha and omega coefficients. Reliability for each alpha and omega was judged against the traditional criteria of a threshold of 0.7 or above meaning good fit for each coefficient, and 0.9 or above meaning excellent fit [32]. The convergent validity of the barriers and benefits scales was examined via correlational estimates of each subscale to external variables. For each pair of correlations, a Fisher’s *Z* score was also calculated to examine relative strength of correlations between the external measure and the barriers scale versus the external measure with the benefits scale. The Fisher’s Z scores were calculated with the Hittner et al. modification of Dunn and Clark’s procedure to adjust for correlated correlations [33].

## Results

### Demographics

The first sample (*n* = 492) was largely male (65.85%), Caucasian (70.2%), college educated (73%), employed full time (80.4%), and in their 30s (M = 32.4, SD = 5.69). The second and third samples comprise random split halves of a larger sample (*n* = 605). The second sample (*n* = 302) was largely female (79.1%), Caucasian (55%), high school educated (56.7%), and in their 20s (M = 24.1, SD = 6.5). Similarly, the third sample (*n* = 303) was largely female (80.2%), Caucasian (56.8%), high school educated (58%), and in their 20s (M = 23.8, SD = 6.1). A summary of demographic statistics are given in Table 1; full demographic statistics are shown in Additional file 1: Table S1.

**Table 1:** Brief demographic statistics for all Samples 1, 2, and 3

	Sample 1 (*n* = 492)	Sample 2 (*n* = 302)	Sample 3 (*n* = 303)
	*M* (*SD*)	*M* (*SD*)	*M* (*SD*)
Age	32.4 (5.69)	24.1 (6.5)	23.8 (6.1)
	% (*n* = 492)	% (*n* = 302)	% (*n* = 303)
Gender (%)			
Male	65.85%	19.87%	18.48%
Female	34.15%	79.14%	80.2%
Non-binary	0%	0.99%	1.32%
Country of residence (%)			
The USA	100%	83.11%	82.84%
The UK	0%	3.97%	2.97%
Australia	0%	7.95%	11.22%
New Zealand	0%	4.97%	2.97%
Race (%)			
White	70.33%	54.97%	56.77%
Hispanic or Latino	5.28%	14.24%	14.52%
Asian	5.69%	14.57%	14.52%
Native American, Alaskan Native, Native Hawaiian or Pacific Islander	0.61%	0.99%	0.99%
Black or African American	16.46%	9.6%	6.27%
Multiple Races/Unknown/Do Not Wish to Disclose	1.63%	5.63%	6.93%

Full demographic statistics are provided in Additional file 1: Table S1

### Item Reduction and Final Scale

#### Parallel Analysis and initial EFA: Sample 1

Parallel analysis [34] of the initial 112 items suggested four factors. The ensuing four factor EFA model was poorly specified, with no primary loadings on the fourth factor. A second EFA was estimated with two factors, to explore the theoretical assumption of one benefits factor and one barriers factor. The two-factor model item loadings reflected one clear ‘benefits’ factor and one clear ‘barriers’ factor. Despite a coherent factor structure, the two-factor EFA model showed poor overall fit with TLI = 0.78. Given the poor overall fit, and the difficulty of interpreting 112 items, item reduction was undertaken.

#### Refinement EFAs and CFAs: Samples 1 and 2

Items were removed in an iterative process considering the following issues: i) high cross-loadings in EFA (cross-loading above a magnitude of 0.2 on multiple factors); ii) loadings below 0.3 in the specified factor in CFA; iii) poor theoretical fit of items.

A resulting two-factor EFA model with 46 items showed a robust factor structure, with factor 1 a ‘barriers’ factor, and factor 2 a ‘benefits’ factor, and no high cross-loadings. The chi-square goodness-of-fit statistic was significant (*X*^2^ = 2281.34, df = 944, *p* < 0.001); however, overall fit was adequate in Sample 1, with a TLI of 0.89, an RMSEA index of 0.054 (90% CI: lower = 0.051, upper = 0.057) and SRMR estimate of 0.04.

A two-factor CFA was estimated based on the prior EFA. The two-factor CFA model showed all 46 items loading significantly. The CFA model showed good overall fit on all indices, except for a significant chi-square goodness of fit: *X*^2^ (988) = 1649.1, *p* < 0.001. The CFI of 0.95, TLI of 0.94, RMSEA of 0.37, and SRMR estimate of 0.062 all showed good fit.

The two-factor 46-item model of the MEX had poor fit in Sample 2 based on a CFI of 0.83, and a TLI of 0.83, prompting further revision. Iterative EFA and CFA estimations were completed after removing items causing poor model-to-data fit in the second sample, resulting in 15 barrier items, and 15 benefit items as the final MEX scale. Two-factor confirmatory factor analysis of the final MEX scale showed good model fit overall in Sample 2, despite a significant chi-square goodness of fit (see Table 3). All items loaded significantly on their respective factors (see Table 2). The inter-factor correlation was negative and moderate in Sample 2, *r* = − 0.479.

**Table 2:** Standardized CFA loadings across the three samples on the 30-item MEX two-factor model

Factor	Items		Sample 1 (*n* = 492)	Sample 2 (*n* = 302)	Sample 3 (*n* = 303)
Benefits	Exercise helps me to have a positive outlook on life		0.549	0.714	0.711
	Exercise makes me feel more self-confident		0.614	0.559	0.672
	Exercising gives me more energy		0.634	0.683	0.783
	Exercise helps me to manage my stress		0.673	0.805	0.795
	Exercise benefits my mental health		0.605	0.722	0.786
	Exercise helps me to feel less angry or irritable		0.588	0.727	0.645
	After exercising, I notice a decrease in my general level of worry and concern		0.588	0.681	0.670
	Regular exercise makes feel me more mentally alert		0.589	0.710	0.704
	Exercise helps me to feel motivated again		0.602	0.626	0.696
	Exercise gives me an instant mood increase		0.598	0.720	0.743
	Exercise makes me feel more energetic		0.651	0.731	0.694
	Exercise helps me to cope with life's stresses		0.660	0.768	0.813
	Exercise helps me manage my mood		0.626	0.782	0.761
	Exercising changes my attitude		0.633	0.668	0.668
	Exercise gives me a long term mood increase		0.521	0.665	0.708
Barriers	I'm too shy or embarrassed to start exercising		0.803	0.759	0.798
	I feel self-conscious about exercising		0.656	0.704	0.762
	I'm worried exercise will hurt		0.749	0.446	0.497
	I'm not confident in my ability to exercise		0.784	0.795	0.808
	Mental exhaustion stops me from exercising		0.762	0.787	0.798
	I worry that I would not be very good at exercising		0.775	0.649	0.618
	Exercising can trigger feelings of anxiety for me		0.786	0.733	0.800
	I feel embarrassed if people see me exercising		0.785	0.486	0.498
	Some days I can't get out of bed, let alone exercise		0.802	0.457	0.425
	I don't want to exercise because I don’t like how my body looks		0.750	0.656	0.657
	I don't want to exercise when I am angry, or in a bad mood		0.721	0.509	0.438
	I worry about what other people think of me exercising		0.727	0.69	0.764
	My exercise or fitness goals seem too hard to achieve		0.662	0.567	0.571
	I've failed to achieve too many exercise goals in the past		0.775	0.614	0.515
	I'm scared of losing my breath while exercising		0.731	0.491	0.513

All loadings statistically significant, *p* < .05

The refined 30-item, two-factor MEX showed good model fit in Sample 1, despite a significant chi-square goodness of fit (see Table 3). All primary item loadings were significant (see Table 2). Inter-factor correlations were weak and negative in Sample 1, *r* = − 0.11.

**Table 3:** CFA fit statistics for the two-factor model of the final 30-item MEX scale in Sample 1, 2, and 3

	CFI	TLI	X2	RMSEA (point)	RMSEA 90% CI (lower)	RMSEA 90% CI (upper)	SRMR
Sample 1	0.951	0.947	679.30*	0.037	0.032	0.042	0.052
Sample 2	0.946	0.942	584.27*	0.039	0.031	0.045	0.056
Sample 3	0.935	0.930	590.36*	0.039	0.032	0.046	0.054

Statistics are robust estimates.

*p < .001, df = 988

#### Confirmation CFA: Sample 3

A two-factor CFA of the 30-item MEX showed overall good fit in the third sample, despite a significant chi-square goodness of fit (see Table 3). All primary item loadings were significant (see Table 2). Inter-factor correlations were moderate and negative, *r* = −0.49.

The final CFA overall fit statistics for each sample can be seen in Table 3. Each row in Table 3 corresponds to the fit statistics of the 2-Factor CFA models in the three samples. Each 2-factor model comprises the 30 final MEX items, as two 15-item factors (benefits and barriers). The fit statistics listed are robust estimates.

### Descriptive Statistics, Reliability, and Convergent Validity

#### The MEX: Descriptive Statistics and Internal Consistency

The means and standard deviations for the MEX barriers and benefits factors in each sample are shown in Table 4. The coefficients alpha [35] and omega [36] were also calculated for each factor in each sample and reflected good internal consistency across each sample and factor, with estimates ranging between 0.900 and 0.951 (see Table 4).

**Table 4:** Means, standard deviations, minimum and maximum, and median scores of the MEX factors as summed total scores

	Sample 1	Sample 2	Sample 3
MEX-benefits			
Mean (SD)	46.8 (6.96)	46.1 (7.41)	45.6 (7.76)
Min/Max score	15–60	27–60	15–60
Median	48	45	45
Cronbach’s alpha	0.900	0.936	0.942
Omega	0.900	0.937	0.943
MEX-barriers			
Mean (SD)	35.8 (11.7)	35.3 (8.73)	35.8 (8.94)
Min/max score	15–56	15–54	15–57
Median	36	36	36
Cronbach’s alpha	0.951	0.906	0.910
Omega	0.951	0.910	0.916

#### The MEX: Convergent Validity

The correlations between the MEX subscales and each of the pre-existing scales are shown in Table 5, along with means and standard deviations of each, and Fisher’s *Z* scores denoting comparative strengths of correlation coefficients between each variable and benefits and barriers, respectively.

**Table 5:** Convergent validity estimates for the MEX benefits and barriers subscales and existing scales

Scale	N	M (SD)	Benefits *r*	Barriers *r*	Fisher’s Z
Sample 1					
MEX-Benefits	492	46.8 (6.96)	–	–	–
MEX-Barriers	492	35.8 (11.7)	0.1*^✝^	–	–
DASS-Depression	466	16.9 (12.6)	0.03	0.725***	12.314***
DASS-Anxiety	457	17.3 (13.7)	0.111*	0.703***	10.51***
DASS-Stress	462	16.9 (11.6)	0.093*	0.707***	10.881***
SWLS	482	22.8 (7.26)	0.271***	0.092*	2.719**
MET·mins/week	344	2972 (2102)	0.276***	0.164**	1.407
SPAF	480	13.2 (3.42)	0.377***	0.097*	4.363***
SDE	492	4.15 (0.782)	0.149**	−0.337***	7.462***
IM	492	4.21 (0.822)	**−**0.018	−0.254***	3.582***
Sample 2/3					
MEX-Benefits	605	45.8 (7.58)	–	–	–
MEX-Barriers	605	35.6 (8.83)	−0.454***	–	–
MASQ-AA	605	28.1 (10.1)	**−**0.033	0.306***	5.003***
MASQ-AD	605	97.5 (12.0)	−0.195***	0.466***	10.086***
MASQ-GDD	605	30.1 (11.1)	−0.174***	0.464***	9.721***
MASQ-GDA	605	23.2 (7.56)	**−**0.107**	0.394***	7.527***
MASQ-GDM	605	38.9 (11.9)	−0.154***	0.459***	9.337***
SPAF	605	10.9 (3.18)	0.437***	−0.606***	16.823***
LTSES	605	0.815 (0.99)	0.335***	−0.451***	11.998***

Correlations significant at *p* < .001 are emphasized in bold

*DASS* Depression Anxiety Stress Scale, *SWLS* Satisfaction with Life Scale, *MET* Metabolic Equivalents of Physical Activity (minutes per week), *SPAF* Subjective Physical Activity and Fitness, *LTSES* Leisure-Time Sport and Exercise Score, *SDE* Self-Deceptive Enhancement, *IM* Impression Management, *MASQ* Mood, Anxiety, and Stress Questionnaire, *AA* Anxious Arousal, *AD* Anhedonic Depression, *GDD* General Distress: Depression, *GDA* General Distress: Anxiety, GDM = General Distress: Mixed

*p < .05, **p < .005, ***p < .001

^✝^Non-parametric relationship

In sample 1, the benefits scale was most strongly associated with satisfaction with life, and subjective ratings of physical activity and fitness; both correlation coefficients were significant and positive. The barriers score for sample 1 was most strongly associated with depression, anxiety, stress, self-deceptive enhancement, and impression management. Depression, anxiety and stress were each positively associated with the barriers scale, whilst self-deceptive enhancement and impression management were negatively associated with barriers to exercise. Physical activity (PA) correlated with each scale with a similar magnitude, both with moderate positive associations.

In sample 2/3, the barriers score was significantly more associated with all variables than was the benefits score. The barriers score was positively correlated with all five MASQ-90 anxiety, depression and stress-related subscales. The barriers score was negatively associated with subjective ratings of physical activity and fitness, and with leisure-time exercise.

#### Barriers and Benefits Association

**Fig. 1:**
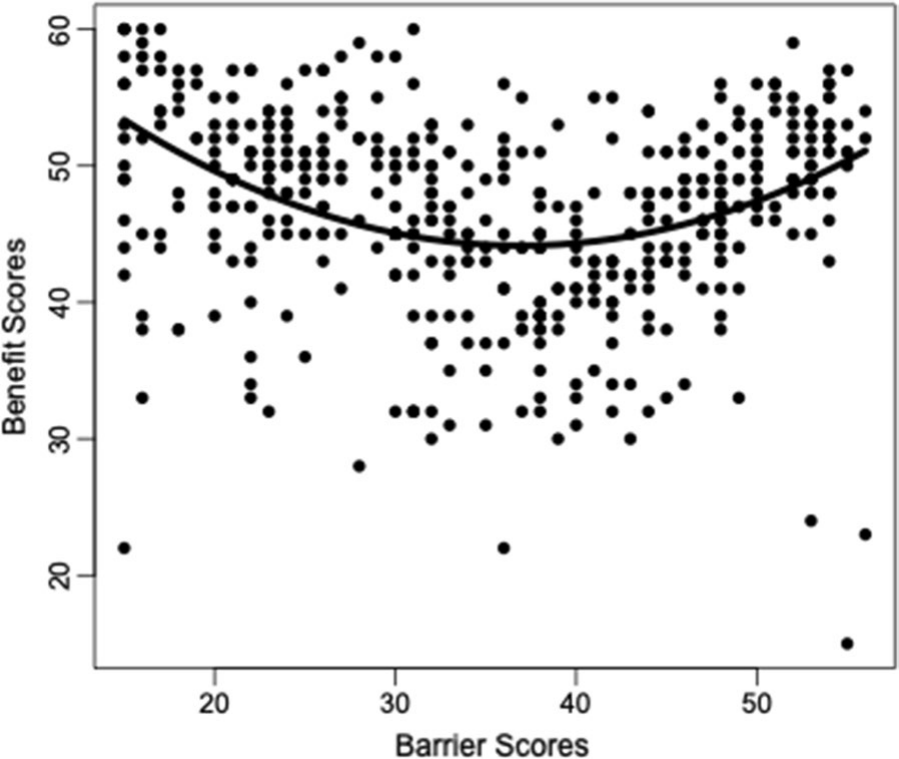
Raw barrier scores by raw benefit scores plotted with curvilinear prediction line

In both data sets, initial inspections of the association between the MEX barrier and benefit subscales were quantified using Pearson’s correlation. The two variables showed a moderate and linear negative correlation in sample 2/3 (*r* = −0.454, *p* < 0.001), but a small positive correlation in sample 1 (*r* = 0.1, *p* = 0.03). Upon further inspection, the relationship between barriers and benefits in sample 1 appeared to be nonlinear (see Fig. 1). A stepwise multiple regression model predicting benefits from the barrier values and the squared barrier values implied a quadratic relationship, F(2, 489) = 34.77, *p* < 0.001, adjusted *R*^2^ = 0.121. Both the barrier values and the squared barrier values were statistically significant predictors of benefits in the stepwise regression model.

#### COVID-19 Impact on Activity and Mental Health

In sample 1, the impact of the COVID-19 pandemic on exercise behaviours was not significantly correlated with neither the MEX benefits (*r* = −0.18, *p* = 0.078), nor the MEX barriers (*r* = −0.03, *p* = 0.759). Similarly, the impact of the COVID-19 pandemic on mental health was not significantly correlated with neither the MEX benefits (*r* = 0.09, *p* = 0.282) nor the MEX barriers (*r* = −0.06, *p* = 0.499).

## Discussion

Results from the present study support the psychometric validity and reliability of the 30-item Mental health-related barriers and benefits to EXercise (MEX) scale in generally healthy adults without severe mental illness. Additionally, the two 15-item subscales (benefits, and barriers) are each well supported psychometrically within the present analysis. Both subscales have high item loadings and high internal consistency, whilst the scale as a whole showed excellent discriminant and convergent validity. The novel MEX scale fills an important gap in the literature regarding the quantification of depression and anxiety-related barriers and benefits to exercise engagement, which are well documented in qualitative research [4, 12], but are lesser known in quantitative research and not yet a key focus of any quantitative measurement of barriers and benefits of exercise.

There was a robust positive association in sample 1 among benefits and total physical activity (PA; i.e. a summation of weekly leisure-time exercise, as well as work, transport, or other non-leisure-time physical activity across an average week). In sample 2/3, there was also a robust positive association between the MEX benefits and leisure-time exercise. These results are important in showing the convergent validity of the MEX in terms of both overall PA, and in leisure-time physical activity, in particular. Similarly, barriers to exercise engagement were robustly positively associated with anxiety- and depression-like symptoms in both data sets and negatively associated with leisure-time exercise in sample 2/3. In both data sets, barriers were significantly more associated with symptoms of affective distress than were the benefits.

Given the number of overall correlations, only those significant at a threshold of *p* < 0.001 are considered in detail. In sample 1, results demonstrated a moderate positive correlation between benefits of exercise and satisfaction with life as predicted. Similarly, benefits of exercise were positively associated with total PA and subjective perception of fitness, suggesting that beliefs about benefits of exercise are related to both actual exercise engagement and the subjective estimates of one’s physical fitness and PA levels. Barriers, on the other hand, were strongly positively correlated with anxiety-like symptoms, depression-like symptoms, and self-reported levels of stress. This result implies that as symptoms of mental ill health increase, so too does one’s experience of mental health-related barriers to exercise engagement. The trend is particularly important to recognize as it further highlights the additional and unique barriers to exercise faced by otherwise generally healthy individuals who experience symptoms of anxiety and depression, as previously examined in qualitative research [4, 12]. To our knowledge, these results mark the first time that the relationships between mental health-related barriers to exercise and symptoms of anxiety, depression and stress have been quantified. Interestingly, there were moderate negative correlations between the barriers scale and both indicators of social desirability biases: impression management and self-deceptive enhancement. These correlations potentially imply that mental health-related barriers to exercise may be underreported by individuals with higher propensity for social desirability biases.

In sample 2/3, the mental health-related benefits to exercise were negatively correlated with symptoms related to anhedonic depression, symptoms related to general distress—depression, and general distress—mixed, as well as symptoms related to general distress—anxiety to a lesser extent. The results would seem to suggest a stronger relationship between benefits and depressive symptoms than benefits and anxious symptoms, though this was not observed in sample 1. Benefits were again positively correlated with the subjective assessment of physical fitness and PA, as well leisure-time exercise and sport engagement, providing important support for the scale’s convergent validity in physical activity measurement and self-perception. The barriers scale was positively correlated with all measures of affective distress symptoms in sample 2/3, where the MASQ-90 was employed. Thus, for each of the five affective distress subscales, higher levels of symptoms corresponded with higher levels of perceived mental health barriers. Additionally, in sample 2/3, the barriers scale was negatively associated with leisure-time exercise, as well as subjective fitness and PA. Thus, higher levels of perceived barriers corresponded with lower levels of exercise, and lower levels of perceived fitness and activity in generally healthy adults without severe mental illness.

Some inconsistency between samples was observed. Firstly, the benefits scale correlated significantly with small and negative effects, with nearly all forms of distress in sample 2/3 except anxious arousal. However, in sample 1, the benefits scale showed only small, positive correlations with anxiety and stress and was not correlated with depression. This particular discrepancy may be due to the distributions of scores of the DASS-42 and the MASQ-90 in data sets 1 and 2, respectively. Whilst the DASS-42 subscales each demonstrated bimodal distributions in sample 1, the MASQ-90 subscales each demonstrated positively skewed distributions in sample 2/3. The benefits subscale scores were negatively skewed in both data sets 1 and 2. Thus, there may be a potential effect of the discrepancies in distributions of anxious and depressive-type symptoms on the reporting of benefits. Future research may seek to include more homogenous clinical samples for a clearer focus of the relationship between reported benefits and mental ill health symptoms.

Secondly, the barriers scale correlated significantly with moderate and negative effects, with both leisure-time physical activity and with subjective fitness in sample 2/3. However, in sample 1, the barriers scale correlated positively but weakly with self-reported metabolic equivalent minutes of PA, and with subjective fitness. Indeed, a key difference arises in the measurement of leisure-time exercise specifically in sample 2/3, compared with using overall PA in sample 1. Given that the questions of the MEX scale ask for self-reported attitudes toward leisure-time exercise behaviours and not all physical activity and movement, it is reasonable to consider the leisure-time exercise scale, as used in sample 2/3, as a better counterpart to the MEX than the measurement of overall PA which was used in sample 1. In consideration of subjective physical activity, it’s worthwhile to note that the distributions of scores differed between data sets. In sample 1, a negatively skewed distribution was observed for subjective physical activity, which coincided with a very low correlation between barriers and subjective activity (*r* = 0.097). Accordingly, previous evidence also suggests a tendency for individuals who are more sedentary to overestimate PA on the IPAQ [22], the scale used in sample 1 only. In sample 2/3, an approximately normal distribution was observed and coincided with a moderate correlation between barriers and subjective activity (*r* = −0.606). Thus, whilst high levels of barriers may be prevalent in individuals who consider their activity and fitness to be low, there may still be a tendency of some individuals to experience higher than expected levels of barriers, despite self-reporting high levels of fitness and physical activity. Such a relationship may be moderated by additional factors, such as affective symptoms, psychological stress, and stages of change for exercise [37], which is a potential area for future exploration. It is worth noting that in some instances, higher levels of self-reported psychological stress has correlated with higher overall PA in young adults [37], but the exact relationship between the MEX barriers scale and differing types of PA beyond leisure-time exercise (e.g. occupational, housework, or transport PA) may still require further examination.

Finally, an additional inconsistency resulted from the relationship between benefits and barriers was inconsistent across data sets. In sample 2/3, a moderate negative linear relationship was found between MEX barrier and benefit scores. However, a moderate curvilinear association was found in sample 1, whereby low levels of barriers and high levels of barriers were both associated with high levels of benefits, but medium levels of barriers were associated with low levels of benefits. Thus, there appears to be potential variability amongst individuals with high levels of mental health barriers to exercise. Previous research has indicated that some, but not all, individuals may increase their overall physical activity in the face of high levels of psychological stress or mental health difficulty, choosing to use physical activity as a form of stress relief [37]. Indeed, the MEX includes stress relief as a theme within multiple items of the benefits scale, potentially explaining why some individuals who report high levels of barriers may also report high levels of benefits. Nevertheless, the source of this variability amongst individuals who strongly endorse the barrier items will be an important focus in future research.

Limitations of the present work warrant consideration. Most importantly, the samples were limited to generally healthy adults without severe mental illness and largely with subclinical symptoms. Whilst the recruitment of generally healthy adults for scale validation was an important first step in validating the MEX, we recognize that the use of clinical samples for validation is crucial to future research with the MEX.

Secondly, comparison between data sets was made difficult by differences in measurement scales, especially for symptoms of depression, anxiety, and stress, and for leisure-time physical activity. Future research using the MEX in separate samples should therefore use consistent measures of depression, anxiety, and stress (i.e. the DASS-42), and leisure-time physical activity (i.e. the LTSES). Additionally, we did not include an objective measure of physical activity. It is important for future work to examine how items on the MEX relate to objective indicators of physical activity, given the typical low reliability found in self-report measures of activity. However, a differentiation may need to be made between leisure-time physical activity and total physical activity in objective measurement. Furthermore, future research should also examine the effects of mental health-related barriers and benefits on separable parameters of exercise prescriptions, including frequency, duration and intensity of exercise, as each parameter may have differing associations with the barriers and benefits scales [38], which may be important in personalization of exercise prescription.

It would also be important for future research to examine the potential effects of individual items or themes of the MEX (e.g. aversive sensations as barriers, or stress relief as a benefit). Due to the factor structure of the scale, whereby subfactors within the barrier and benefit factors could not be established in this study, conclusions about individual items or themes could not be reached. Future research could examine individual differences across MEX items, as certain items or themes may be of particular importance for individualization of treatment.

Finally, the sampling was limited to mostly Caucasian, English-speaking participants from western countries, with mostly subclinical depressive and anxious symptoms. Thus, ascertaining norms and factorial invariance for the MEX across diverse sociocultural samples, and in clinical samples, is an important consideration for future research.

## Conclusions

We have developed a 30-item scale of mental health-related benefits and barriers to exercise, comprising 15 barriers and 15 benefits. The aim of this research was to produce a quantitative measurement of the bidirectional relationship between transdiagnostic symptoms of mental health disorders and physical exercise, which can be used at both the individual level in clinical settings, and at broader levels for research. The present research provides evidence that scores on the MEX are valid and reliable in generally healthy adults without severe mental illness, as highlighted by the MEX subscales meeting excellent standards of internal consistency, and factorial, convergent, and discriminant validity. We believe that due to these properties, as well as the brevity and breadth of the scale, the MEX will be a useful measurement tool in both clinical and research settings for the mental health-related barriers and benefits to exercise.

## Supplementary Information

**Additional file 1:** Supplementary demographic information, and the data cleaning procedure for each of the samples analysed.

## Data Availability

Data are available online: https://osf.io/dm3kz/. The MEX scale is available online: https://osf.io/gnqfr/wiki/home/. Analysis scripts are available upon request to the corresponding author. The availability of other materials as described in the Methods section will be at the discretion of the copyright holders per each item.

## Abbreviations

MEX: The Mental health-related barriers and benefits to EXercise scale
DASS-42: Depression Anxiety Stress Scale
SWLS: Satisfaction with Life Scale
METs: Metabolic Equivalents of Physical Activity
SPAF: Subjective Physical Activity and Fitness
LTSES: Leisure-Time Sport and Exercise Score
SDE: Self-Deceptive Enhancement
IM: Impression Management
MASQ: Mood, Anxiety, and Stress Questionnaire
MASQ-AA: Anxious Arousal
MASQ-AD: Anhedonic Depression
MASQ-GDD: General Distress: Depression
MASQ-GDA: General Distress: Anxiety
MASQ-GDM: General Distress: Mixed
PA: Physical Activity
IRV: Intra-individual response variability
EFA: Exploratory Factor Analysis
CFA: Confirmatory Factor Analysis
TLI: Tucker–Lewis Index
CFI: Comparative fit index
SRMR: Standardized Root-Mean-Squared Residual
RMSEA: Root-mean-square error of approximation
