# Supplementary material for: Development and Psychometric Validation of the Mental Health-Related Barriers and Benefits to EXercise (MEX) Scale in Healthy Adults
Source: Sports Med Open. 2023 Feb 23;9:18. doi: 10.1186/s40798-023-00555-x (PMC9947889; doi:10.1186/s40798-023-00555-x)
Supplement: Supplementary file 1 — Additional file 1: Supplementary demographic information, and the data cleaning procedure for each of the samples analysed. [file 40798_2023_555_MOESM1_ESM.docx]

**Supplemental Materials:**

**Article Title:** Development and psychometric validation of the Mental health related barriers and benefits to EXercise (MEX) scale in healthy adults

**Journal:** Sports Medicine – Open

**Authors:** Madeleine L. Connolly*^1^, Stephen C. Bowden^1,2^, Michaela C. Pascoe^3^, & Nicholas T. Van Dam^1^

^1^Melbourne School of Psychological Sciences, The University of Melbourne, Melbourne, Australia, 3010;

^2^Centre for Clinical Neurosciences & Neurological Research, St Vincent’s Hospital Melbourne, Melbourne, Australia, 3065;

^3^ Institute for Health and Sport, Victoria University, Melbourne, Australia, 3011

**Corresponding Author email:** mconnolly@student.unimelb.edu.au

**Supplemental Material A**

**Table S1.**

*Demographic statistics for all Samples 1, 2 and 3*

|  | **Sample 1 (n=492)** | **Sample 2 (n=302)** | **Sample 3 (n=303)** |
| --- | --- | --- | --- |
|  | *M* (*SD*) | *M* (*SD*) | *M* (*SD*) |
| **Age** | 32.4 (5.69) | 24.1 (6.5) | 23.8 (6.1) |
|  | % (n = 492) | % (n = 302) | % (n = 303) |
| **Gender (%)** |  |  |  |
| Male | 65.85% | 19.87% | 18.48% |
| Female | 3.25% | 79.14% | 80.2% |
| Non-binary | 0% | 0.99% | 1.32% |
| **Country of Residence (%)** |  |  |  |
| USA | 100% | 83.11% | 82.84% |
| United Kingdom | 0% | 3.97% | 2.97% |
| Australia | 0% | 7.95% | 11.22% |
| New Zealand | 0% | 4.97% | 2.97% |
| **Race (%)** |  |  |  |
| White | 70.33% | 54.97% | 56.77% |
| Hispanic or Latino | 5.28% | 14.24% | 14.52% |
| Asian | 5.69% | 14.57% | 14.52% |
| Native American, Alaskan Native, Native Hawaiian or Pacific Islander | 0.61% | 0.99% | 0.99% |
| Black or African American | 16.46% | 9.6% | 6.27% |
| Multiple Races/Unknown/Do Not Wish to Disclose | 1.63% | 5.63% | 6.93% |
| **Education level (%)** |  |  |  |
| High School or Less | 7.52% | 23.18% | 24.09% |
| Some College/Completed 2-Year College | 19.31% | 33.11% | 33.99% |
| 4-Year College | 41.46% | 28.81% | 30.69% |
| Graduate Degree | 31.5% | 14.57% | 11.22% |
| **Employment Status (%)** |  |  |  |
| Full-time employment | 83.94% | 26.82% | 29.37% |
| Part-time employment | 9.35% | 29.8% | 34.65% |
| Unemployed | 6.3% | 43.38% | 35.64% |
| Retired | 0.2% | 0% | 0% |
| Not working due to disability | 0.2% | 0% | 0.33% |
| **Household Income (%)** |  |  |  |
| $0 - $25,000 | 8.94% | 24.5% | 18.81% |
| $25,001 - $50,000 | 33.74% | 23.18% | 28.38% |
| $50,001 - $100,000 | 47.15% | 29.8% | 32.67% |
| $100,001 - $249,000 | 9.96% | 19.87% | 17.49% |
| $249,001+ | 0.2% | 2.65% | 2.64% |
| **Marital Status (%)** |  |  |  |
| Married or living as married | 67.07% | 20.86% | 16.17% |
| Separated, Divorced, Annulled or Widowed | 5.08% | 0.99% | 0.33% |
| Never been married | 27.85% | 78.15% | 83.5% |

##

**Supplemental Material B**

## Full Description of Data Cleaning and Preparation

## *Sample 1.* The participants in Sample 1 were recruited via Mturk (total N = 1109). Of the 1109 cases, data from 505 (45.4%) participants was removed manually for unsatisfactory or failed recaptcha scores (n = 66), attention checks (n = 51), and written prompts (n = 321), or low total completion rates (n = 67).

The 16-item BIDR scale was chosen for data processing, as the scale has good psychometric properties [(Hart et al., 2015)](https://www.zotero.org/google-docs/?J8SDi8), and each of the 16 items exhibited normal distributions in this dataset. Of the 604 cases remaining, cases were removed if they were missing data from all of the BIDR items (n = 21). Following the removal of missing data, cases were removed for the following: long string responses over the threshold of 6 (n = 29), IRV estimates of above the cut-off threshold of 2.5 (n = 45), and Mahalanobis distance estimates above the threshold of 32.25 (n = 17). The final dataset comprised 493 participants.

***Samples 2 and 3.*** The participants in Sample 2 and 3 were recruited via Prolific (total N = 767). Of the original 767 cases, data from 53 (6.9%) participants was removed manually for unsatisfactory or failed recaptcha scores (n = 7), attention checks (n = 8), or written prompts (n = 27), or for low total completion rates (n = 11).

The full MASQ-90 scale was chosen for data processing, as the scale has good psychometric properties. Of the 714 cases remaining after manual data cleaning, no cases showed any missing data from any MASQ-90 items. Following missingness analysis, cases were removed for the following: long string responses above the threshold of 16 (n = 5), IRV scores above the cut-off of 1.55 (n = 40), and Mahalanobis distance estimates above the cut-off score of 137.2 (n = 64). The final dataset comprised 605 participants in total, which was then split randomly into two halves to form Sample 2 (n = 302) and Sample 3 (n = 303).
